# Diurnal variation in the production of vocal information about food supports a model of social adjustment in wild songbirds

**DOI:** 10.1098/rspb.2018.2740

**Published:** 2019-02-27

**Authors:** F. Hillemann, E. F. Cole, S. C. Keen, B. C. Sheldon, D. R. Farine

**Affiliations:** 1 Edward Grey Institute of Field Ornithology, Department of Zoology, University of Oxford, Oxford OX1 3PS, UK; 2 Bioacoustics Research Program, Cornell Laboratory of Ornithology, Ithaca, NY 14850, USA; 3 Department of Collective Behaviour, Max Planck Institute for Ornithology, 78464 Konstanz, Germany; 4 Chair of Biodiversity and Collective Behaviour, Department of Biology, University of Konstanz, 78464 Konstanz, Germany; 5 Centre for the Advanced Study of Collective Behaviour, University of Konstanz, 78464 Konstanz, Germany

**Keywords:** collective animal behaviour, foraging, group living, signalling, social information use, vocal recruitment

## Abstract

Wintering songbirds have been widely shown to make economic foraging decisions to manage the changing balance of risks from predation and starvation over the course of the day. In this study, we ask whether the communication and use of information about food availability differ throughout the day. First, we assessed temporal variation in food-related vocal information produced in foraging flocks of tits (*Paridae*) using audio recordings at radio-frequency identification-equipped feeding stations. Vocal activity was highest in the morning and decreased into the afternoon. This pattern was not explained by there being fewer birds present, as we found that group sizes increased over the course of the day. Next, we experimentally tested the underlying causes for this diurnal calling pattern. We set up bird feeders with or without playback of calls from tits, either in the morning or in the afternoon, and compared latency to feeder discovery, accumulation of flock members, and total number of birds visiting the feeder. Irrespective of time of day, playbacks had a strong effect on all three response measures when compared to silent control trials, demonstrating that tits will readily use vocal information to improve food detection throughout the day. Thus, the diurnal pattern of foraging behaviour did not appear to affect use and production of food-related vocalizations. Instead, we suggest that, as the day progresses and foraging group sizes increase, the costs of producing calls at the food source (e.g. competition and attraction of predators) outweigh the benefits of recruiting group members (i.e. adding individuals to large groups only marginally increases safety in numbers), causing the observed decrease in vocal activity into the afternoon. Our findings imply that individuals make economic social adjustments based on conditions of their social environment when deciding to vocally recruit group members.

## 1. Introduction

Many species demonstrate strong temporal behavioural patterns over the course of the day in response to environmental changes such as photoperiod, temperature, food availability, and predation risk [1–3]. Energy management and diurnal foraging patterns depend on an individual's current physiological state, as well as their past and anticipated state [4,5]. For example, foraging activity throughout the day in small wintering songbirds is influenced by the inherent trade-off between risk of starvation and risk of predation; birds must gain enough fat during the day to survive through the night, but this is balanced by the increased predation risks associated with weight gain and impaired mobility [6–8]. Birds' diurnal changes in body fat, which can be used as a proxy for foraging activity, generally show a sharp increase in mass during early morning hours, to compensate for overnight body fat loss, and another increase in body mass during the hours immediately before roosting [6,7]. The hypothesis that wintering songbirds should delay accumulating fat during the day to lower predation risk suggests that their foraging tactics, and thus their behaviour, should change as their primary concern shifts from avoiding predators to surviving the night ahead. In accordance with this suggestion, birds from winter foraging flocks appear to apply different foraging strategies over the course of the day, prioritizing the detection of food patches in the morning, and exploiting these known patches later in the day, when the cost of exploration is high relative to foraging on a previously identified food source [9].

When searching for food sources, animals can use information generated by the behaviour of others (‘social information’), which is usually less costly and faster to acquire than information about the distribution of resources through direct, trial-and-error-based interactions with the environment (‘personal information’; [10,11]). However, social information can be less reliable than personal information and might result in sub-optimal behaviour, especially in rapidly changing environments [12–15]. Thus, predictions derived from theoretical models and empirical studies suggest that individuals should rely on social information when personal information cannot be gathered reliably and at a low cost [13,16]. On the other hand, when both types of information are available but in conflict, personal information should be preferred and conflicting social information ignored ([14,17]; but see [18] and [19] for examples of conformist social learning). If availability and reliability of personal information improves with time, owing to accumulated personal experience, animals should reduce their reliance on social information and instead rely on personal information later in the day. Whether the use of food-related social information has such temporal components, shifting over the course of a day in relation to foraging strategies, has yet to be explored.

A parallel decision that some animals appear to make when foraging is whether to actively produce information about the presence of food for others. In many species, vocalizations given upon food discovery attract conspecific and heterospecific foragers, functioning as a form of social recruitment [20]. Recruiting others to a food source provides a range of potential benefits to the caller, including reduced predation risk [21,22], increased inclusive fitness via kin selection [23,24], increased mating opportunities [25,26], or the ability to cooperatively defend the resource [27,28]. However, these benefits diminish with an increase in competition as group size increases, and studies have suggested that individuals make different decisions based on whether recruiting others will lead to an increase in competition [29]. Moreover, calling is a conspicuous behaviour that facilitates prey detection and localization for predators [30,31]. For example, sparrowhawks, *Accipiter nisus*, attacked stuffed models of crested tits, *Parus cristatus*, significantly more often when presented along with playbacks of the species' long-distance calls, compared to when the model was displayed without playbacks [32].

Given these considerations, we expect that the extent to which individuals produce vocalizations which attract group members to a food source (‘recruitment calls’), depends not only on levels of competition and predation risk, but also on how the social environment mediates the trade-off between these factors. To understand the interaction between these components, we outline an economic framework that predicts under which social conditions individuals should and should not produce such recruitment calls. First, a solitary forager is expected to produce recruitment calls because it conveys large benefits with regard to relative predation risk, at a comparably small cost of competition: recruiting just one individual will halve the risk of predation, while competition will not be greatly increased if the food resource is shareable. Further, the probability of being heard by another individual or group is high (because there are *N* − 1 potential listeners, where *N* is the local population size), whereas the probability of a predator attack is low (for the same reason, that there are *N* − 1 other individuals located elsewhere that could be detected). By contrast, the propensity to vocally recruit group members to a food source should reduce with an increase in foraging group size. An individual that forages in a large group would receive only a slight increase in anti-predator benefits by recruiting an additional group member (because 1/*N* is nonlinear, and 1/(*N* + 1) is only marginally larger than 1/*N* when *N* is ≫ 1), but might suffer from a disproportionate increase in the within-group competition for food. The exact relationship between group size and competition between group members depends on the shareability of the food, while the costs and benefits associated with an increase in foraging group size also depend on an individual's position within the group; access to food and anti-predator effects are higher for dominant individuals in the centre of the group [33–36]. In table 1 we outline the relative costs and benefits of making recruitment calls under different social conditions, allowing us to make predictions about when recruitment calling is expected. Using this framework, we predict that individuals will produce recruitment calls when foraging in a larger group (relative to the current group size) yields a net benefit to the caller.

**Table 1.** Cost–benefit considerations for recruitment calling. (Framework highlighting the shifting costs and benefits associated with competition and safety from predators. When costs are lower than benefits (e.g. when alone or in a small group), individuals should make recruitment calls. By contrast, when individuals are in large groups, adding more individuals to the group will increase competition but provide fewer added benefits, in which case an individual should not recruit. Classification of effects as high or low should be considered in relation to other scenarios.)

potential caller's social context	effect of additional group member (GM)	cost–benefit balance for recruitment calling
alone	very high probability attracting GM	cost ≪ benefit
	low competition between GM	
	very high value regarding predation risk	
	low probability of predator attack	
small group	high probability attracting GM	cost = benefit
	increasing competition between GM	
	low value regarding predation risk	
	low probability of predator attack	
large group	low probability attracting GM	cost > benefit
	high competition between GM	
	low value regarding predation risk	
	high probability of predator attack	

Here, we propose, and experimentally test, two hypotheses that may lead to temporal variation in the production and use of food-related acoustic information. The ‘economic hypothesis’ assumes that individuals experience a shift in the costs and benefits of calling owing to a change in group size (as outlined in table 1), which causes a producer-driven reduction of vocal information with increasing foraging group size. Accordingly, if group sizes increase throughout the day, it becomes less beneficial to recruit additional individuals to the foraging group, and consequently fewer calls will be produced later in the day. Alternatively, the ‘foraging strategies hypothesis’ assumes that a switch in foraging strategies over the course of the day (as demonstrated in [9]) causes individuals to change from using social information in the morning (to find food) to ignoring social information in the afternoon (once personal information about the environment has been accumulated). This hypothesis predicts that call production is reduced in the afternoon as it is no longer effective at recruiting others.

In this study, we test these competing hypotheses using data from mixed-species foraging flocks of tits (*Paridae*). First, we establish how calling patterns in mixed tit flocks vary throughout the day using audio recordings at feeding stations. Next, we explore the underlying causes of temporal variation in calling behaviour. To do so, we set up bird feeders with or without a playback of calls from tit species, either in the morning or in the afternoon, and measured latency until discovery and number of birds visiting the feeder. Given our knowledge of the daily patterns of foraging in wintering songbirds, we can predict that the decisions to produce information about food should also differ throughout the day, mirroring changes in birds' foraging behaviour and social environment. Under the economic hypothesis, we would expect that individuals vocalize less in the afternoon, when group sizes are large and vocalizations might attract predators, but that listeners will respond to experimentally presented calls irrespective of time of day. Under the foraging strategy hypothesis, we would predict that flock members can be attracted by calls given in the morning but not in the afternoon, resulting in a listener-driven reduction in calling over the course of the day.

## 2. Methods

### (a) Study system

The study was conducted in Wytham Woods, Oxford, UK (51°46′ N, 1°20′ W), on a wild population of individually tagged Parid species (blue tit, *Cyanistes caeruleus*; great tit, *Parus major*; marsh tit, *Poecile palustris*). Birds were tagged either as nestlings or as adults, when caught at nest-boxes, or using mist-nets in winter (percentage of the population being tagged was estimated at *ca* 70–80% for the winter seasons 2011–2014: [37]). Each bird was fitted with a unique British Trust for Ornithology (BTO) metal leg ring, and a plastic leg ring carrying a passive integrated transponder (PIT tag, IB Technologies, UK). PIT tags can be read by radio-frequency identification (RFID) antennas attached to bird feeders (Dorset ID, Netherlands), providing a timestamp of feeder visits.

Tits in this population are almost always found in mixed-species flocks [38,39], and information is readily transferred across species [40,41]. Thus, we analyse our data at the community level. However, we also report analyses of species-level patterns in the electronic supplementary material for two main reasons: first, because marsh tits are food-caching birds, they could express different behavioural strategies to blue tits and great tits (cf. [42]). Second, not all species are equally represented in the population, with great tits being most numerous across all experimental sites in this study (mean ± s.d.: 50.1 ± 14.2%), followed by blue tits (36.0 ± 10.6%), whereas marsh tits (14.0 ± 10.3%) are scarcer despite being found throughout the woodland.

### (b) Measuring diurnal variation in vocalizations at feeding stations

Over the course of two winters, we ran 18 independent feeder discovery trials at unique sites across the study area (10 trials in February to March 2016, eight trials in February to March 2017). At each site, we deployed feeding stations after sunset on the evening before the trial to ensure natural discovery of the novel feeder the next morning (following [9,40,43]). Feeders were filled with sunflower seeds, and RFID antennas automatically recorded the time and identity of PIT-tagged birds for each feeder visit. We recorded vocalizations at each feeder throughout the length of the trial, i.e. from sunrise to sunset, using small voice recorders with omnidirectional microphones (VN-741 PC Digital Voice Recorder, Olympus, Japan) attached to the feeder.

### (c) Playback experiments

#### (i) Experimental procedure

We conducted playback experiments and control trials at 12 sites during the non-breeding season in February and March 2017. The discovery and experimental sites were distinct, widely distributed across the study area (separated by ≥200 m), and had never previously had feeders present. We used a fully balanced experimental design, with each site used for four conditions in random order: playback morning (AM), control AM, playback afternoon (PM), control PM. Morning experiments were run from 08.30 onwards, and afternoon experiments from 14.30. Playbacks were broadcast at 72 ± 3 dB (measured with a Maplin, UK, ST-85C sound level meter at 1 m distance), using a loudspeaker (Road Rocker™, Ion Audio LLC., USA) connected to a Raspberry Pi (Raspberry Pi Foundation, UK), and set up on a platform of 2 m height, approximately 3 m from the feeder. By running control trials using a silent dummy loudspeaker, we account for potential effects on feeder discovery owing to differing activity levels, foraging strategies, or flock sizes in the morning and the afternoon (see [9]). For each trial, we temporarily deployed a bird feeder providing sunflower seeds, and recorded latency until feeder discovery and number of birds visiting. Feeder visits of PIT-tagged birds (time and identity) were recorded automatically through RFID antennas attached to the feeder, and trials were additionally video recorded. Trials started with the delayed, automated onset of the playback, or after an equal delay in the control trials, when the experimenter finished setting up the equipment and left the site. While setting up the experiment, no bird could be seen or heard at the experimental site. Each trial lasted for 120 min and feeders were removed quickly afterwards. We waited a minimum of two days (mean ± s.d.: 4.6 ± 2.09 days) after a feeder was discovered before conducting the next trial at that given site.

#### (ii) Stimulus design

Previous work in our system showed that any of the species (blue tits, great tits, and marsh tits) can be the first to discover food sources [9,39,41,44]. Therefore, we created mixed-species stimuli by combining chirp calls of blue tits, chaffinch-like chirp calls of great tits, and chick-a-dee-like dä/D calls of marsh tits (classification and naming of call types following [45], figure 2). Audio files and spectrograms of the call types can be found in the electronic supplementary material, figure S4.

Food-recruitment calls have been described for closely related species (black-capped chickadees, *Parus atricapillus*: [46]; Carolina chickadees, *Poecile carolinensis*: [47]; willow tits, *Poecile montanus*: [48]), but there is no evidence that the species concerned in our study make distinct food-related recruitment calls. However, their calls adapt across a variety of social contexts, including flock movement and foraging [45,49], and specifically the number of notes included in a call appears to provide context-dependent information. For example, dä/D calls of marsh tits given in predator demonstration trials have more notes than dä/D calls recorded during foraging [45]. For the playback stimuli, we used calls that consisted of mean ± standard deviation (s.d.): 3.1 ± 1.3 notes, reflecting the characteristics of calls naturally occurring in a foraging context for this population (mean number of chirp notes in blue tit and great tit calls: 2.4 ± 1.7 s.d., mean number of dä/D-notes in marsh tit calls: 2.6 ± 1.8 s.d.; analysis of 577 chirp calls and 65 dä/D calls recorded at feeding stations during the first phase of this study; see the electronic supplementary material, table S1 for mean number of notes per call recorded during predator presentation experiments, data from [45]).

We selected calls from high-quality recordings of natural calling sequences that were published under a Creative Commons license on www.xeno-canto.org, an online archive of bird calls and birdsongs. For editing the recordings and generating stimulus files, we used Audacity^®^ recording and editing software [50]. Recordings were bandpass-filtered to reduce low-frequency background noise, and we normalized volume levels to peak-amplitude. We created twelve unique 10 min stimuli (one for each experimental site), by combining four calls from each species in randomized order. The stimulus was presented twice during an experimental session, once at the start of the trial, and once one hour after the onset of the first presentation. For a schematic of the stimuli see the electronic supplementary material, figure S1.

### (d) Data analysis

#### (i) Audio recording at feeding stations

Recordings were manually inspected for calls from tits using sound analysis program PRAAT [51]. We only considered calls given within very close proximity to the feeder and immediately before or after a bird landed on the feeder (which was detectable because landing on the feeder was audible on the recording). Following Carlson *et al*. [45], we classified call types based on structural differences that are clearly visible in the spectrograms. We summarized the number of calls given for each hour of daylight, the number of individual birds visiting the feeder during that hour, and the total number of feeder visits per hour for each site, and calculated mean values and standard errors for all measures across all trials (*n* = 18). We then compared the number of calls made per visit for each hour of the day using the same permutation test as Farine & Lang [9]. We first calculated the difference between the mean calls per visit for morning hours (before 12.00) to the mean calls per visit for afternoon hours (after 12.00). We then compared this observed difference to a distribution of 10 000 differences generated by randomizing the ‘hour’ column in our dataset (restricted within trial). The observed difference was significant if it was larger than 95% of the differences generated by the randomized datasets [52].

#### (ii) Playback experiment

Feeders were usually discovered within one hour of deployment in the majority of trials (in 38 of 48 trials; in 7 of 48 trials the feeder remained undiscovered during the 2 h deployment). Thus, we analysed behavioural responses to the experimental treatments during the first hour of the trial. We measured latency to feeder discovery, initial recruitment (number of individuals logged at the feeder within the first two minutes of discovery), and total number of unique individuals (using PIT-tag data) that visited the feeder over the course of the trial (60 min). Latency to feeder discovery was measured as the time elapsed between the onset of the trial and the first recorded visit to the feeder. Video recordings were used to ensure we had accurate estimates of the time that the first bird recorded automatically was indeed the first individual taking a seed across all trials. When no bird visited the feeder within the first hour of the trial (*n* = 10), we censored the latency by setting it to 60 min. Owing to technical problems with the automated logging of tagged birds in five trials, we used a restricted dataset (*n* = 43) for analysing the number of birds visiting the feeder, both during the initial recruitment and the 60 min trial.

The balanced design of the playback experiment allowed us to test our hypothesis regarding the use of social information across the day, by contrasting responses to the same social stimulus in two contexts (morning/AM and afternoon/PM). To estimate the effect of time of day (AM, PM) and experimental treatment (playback, control) on the three response measures, we used generalized linear mixed models (GLMMs) with gamma distribution to overcome over-dispersion in skewed latency data [53], and Poisson error distribution in models with count values (i.e. number of birds). As the effect of playbacks might have been different for morning and afternoon trials, we included an interaction term (time of day * treatment) as fixed effect. However, for simplification of the final models, we removed nonsignificant interactions [54]. We included trial order (i.e. experimental day, 1–4) as a main effect to account for any habituation over the course of the trials. To factor in non-independence of repeated trials at the same site, we included experimental site as a random effect in all models. We additionally conducted all three models analysing birds' response measures separately for each species, using the same methods as described above on species-specific subsets of the data. All tests were two-tailed and significance levels set at 0.05, and models were fitted in R (v. 3.1.2; [55]) using the glmer function in the lme4 package [56].

## 3. Results

### (a) Diurnal patterns of vocal and foraging activity at feeding stations

**Figure 1.**
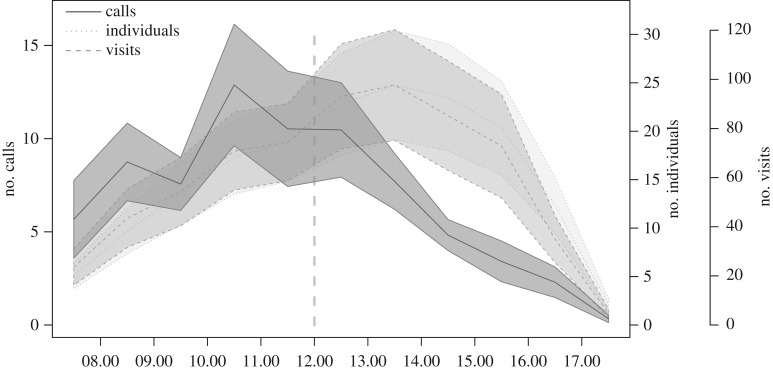
Diurnal patterns at feeders. Mean number of calls given per hour (solid line), and mean number of individuals and feeder visits recorded at a feeder (dotted and dashed line respectively). Shaded areas are standard errors of means, across all 18 sites. Data on number of calls show a clear divergence with number of visits after 12.00 (vertical line).

Feeders were usually discovered within the first hours after sunrise (median [first quartile, third quartile]: 27 [13.5, 60.8] minutes after sunrise; times for sunrise were extracted from www.timeanddate.com). Vocal activity at these feeders was highest during the morning hours and then decreased throughout the day, whereas the number of individuals and the number of feeder visits increased into the afternoon, peaking at about 13.00, and then decreasing into the evening, presumably as individuals departed for roosting in the evening (figure 1). We found that birds made significantly more calls per visit in the morning hours than in the afternoon hours (PM-AM: −0.24, *P*_rand_ < 0.001, 95% range of differences from randomized data: −0.11, 0.10; see also the electronic supplementary material, figure S2). We provide additional details on the diurnal patterns of vocal activity in the electronic supplementary material, including temporal pattern and spectrograms of the main call types recorded (electronic supplementary material, figures S2–S4). Although we ran 18 replicates within the same woodland (10 trials in 2016, eight trials in 2017), the majority of individuals were observed at only one trial (electronic supplementary material, figure S5).

### (b) Experimental test of the hypotheses

#### (i) Latency to feeder discovery

**Figure 2.**
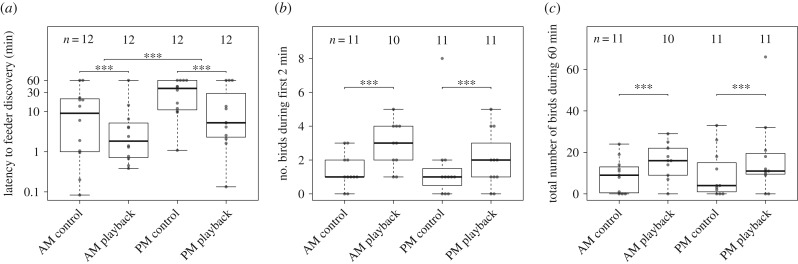
Responses to the four experimental treatments: (*a*) latency to feeder discovery (note log scale), (*b*) initial recruitment, and (*c*) number of birds visiting during the first hour of the trial. Boxplots show the median and interquartile range (IQR), whiskers represent 1.5 × IQR. Dots represent individual trials, number of trials included in the analysis is given for each experimental treatment respectively. Starred bars indicate significant differences (*p*-values <0.001) between treatments based on outcomes of the GLMMs.

Feeders were discovered significantly more quickly in the playback treatment than in control trials (table 2); compared to the control, latency to feeder discovery was on average five times shorter with a playback in morning trials (AM control median: 9.8 min, IQR: 19.81 min; AM playback median: 1.9 min, IQR: 4.60 min; figure 2*a*), and about seven times shorter in afternoon trials (PM control median: 37.85 min, IQR: 48.94 min; PM playback median: 5.25 min, IQR: 34.42 min; figure 2*a*). Irrespective of playback treatment, time to discovery was generally shorter when feeders were deployed in the morning than in the afternoon (table 2). The chronological order of trials had a comparatively low, reversed, effect on the latency: latencies were longest in first trials and shorter when birds had previously used a feeder at that site (table 2), and the effect size was approximately half that of the experimental treatment variables.

**Table 2.** Results of generalized linear mixed models. (Summary of estimated effects on the three response variables describing feeder discoveries, by the fixed effects of time of day (PM relative to AM), treatment (playback relative to silent control), and order of trial at a given site (1st, 2nd, 3rd, or 4th). Experimental site was included as a random term in all models. The degrees of freedom (d.f.), coefficient, standard error (s.e.), *z*-statistic and standard *p*-value are provided.)

	response variable			
fixed factors	d.f.	coefficient ± s.e.	*z*	*p*
latency first bird				
intercept	1	8.03 ± 0.45	17.92	<0.001
time of day	1	1.03 ± 0.30	3.40	<0.001
treatment	1	−1.17 ± 0.31	−3.74	<0.001
order of trial	1	−0.59 ± 0.13	−4.58	<0.001
initial recruitment				
intercept	1	1.25 ± 0.23	5.41	<0.001
time of day	1	−0.09 ± 0.09	−0.97	0.33
treatment	1	0.52 ± 0.09	5.80	<0.001
order of trial	1	0.31 ± 0.04	7.51	<0.001
number of birds				
intercept	1	1.16 ± 0.28	4.18	<0.001
time of day	1	−0.09 ± 0.09	−1.06	0.29
treatment	1	0.53 ± 0.09	5.88	<0.001
order of trial	1	0.31 ± 0.04	7.37	<0.001

#### (ii) Initial recruitment

The number of individuals that visited the feeder in the first 2 min after discovery was significantly greater for the playback treatment than for the control treatment (table 2; figure 2*b*), but initial group sizes did not differ between feeders discovered in the morning and those discovered in the afternoon (table 2). Over the course of the experiment, when multiple trials had been conducted at a given site, more individuals were recorded in the first 2 min period, as indicated by a positive effect of trial order (table 2).

#### (iii) Number of birds

The total number of unique individuals recorded at a feeder over the first hour of the trial was significantly higher in playback trials than in control trials (table 2; figure 2*c*). This pattern was unaffected by time of the day, i.e. the number of unique individuals did not differ significantly between morning and afternoon trials (table 2). Order of trials on the number of unique individuals again had comparatively small effect: subsequent trials had higher numbers of individuals visiting the feeder than first trials (table 2). Despite running four treatments in each of the 12 replicated sites, nearly 80% of all individuals were only ever detected at a single site (electronic supplementary material, figure S5).

#### (iv) Species-level patterns

Species-level analyses of the responses to playbacks revealed nearly identical results to the composite results (electronic supplementary material table S2 and figure S6). In all three species, the direction of the effect of treatment had the same direction with similar effect sizes. Because the marsh tit population was much smaller, owing to their lower relative abundance in the population, the effect sizes for the number of recruits (initial and total) are lower than for blue tits and great tits, although the difference may be less than expected given the difference in population sizes. The effect of time of day on latency to arrive appears to be stronger for blue tits than for other species.

## 4. Discussion

We present data revealing diurnal dynamics of vocal behaviour and foraging activity in wintering mixed-species foraging flocks of tits, and experimentally test predictions from two hypotheses about the underlying causes. By recording vocalizations given at feeding stations over the course of the day, we find clear temporal variation in call rate; vocal activity was highest in the morning and decreased in the early afternoon. By contrast, group size continued to increase until late in the day. In the second part of the study we experimentally evaluate the recruitment function of these calls, and whether their value changes over the course of the day. Our playback experiment shows that social information results in a faster discovery of resources and rapid accumulation of individuals at food patches. This suggests that calls given at the feeders indeed have a recruitment function similar to food-associated vocalizations in other species (reviewed in [20]), including closely related Parid species [46–48]. However, by using a balanced experimental design, our results also show that birds use social information about novel food patches equally throughout the day. Thus, our data support the economic hypothesis, which predicts that individuals reduce calling in the afternoon owing to the reduced benefits of recruitment, and not the foraging strategies hypothesis, which suggests that birds stop calling in the afternoon because social information is ignored.

The notion that birds will reduce their propensity to recruit flock members with increasing group size has been reported in other avian species on a much smaller timescale, analysing changes in calling behaviour as flocks establish at bird feeders. Calling rate at feeders was found to be inversely proportional to foraging flock size in house sparrows, *Passer domesticus* [29] and willow tits [48], and in Carolina chickadees, chick-a-dee calls given by the first bird to arrive at a new food source contain more D-notes and were found to attract conspecifics faster than calls of the subsequent individuals [47]. Spider monkeys, *Ateles geoffroyi*, adjust their food calling not only to the number of conspecifics present but to their social status, and thus manipulate the size and social composition of foraging subgroups [57]. Together, these studies provide additional support for the economic hypothesis for recruitment, suggesting that the decisions to make recruitment signals are subject to the social environment, and specifically group size.

Our balanced playback experiments enabled us to directly compare birds' responses to standardized stimuli at different times of the day, and allowed us to contrast the economic hypothesis against an alternative hypothesis that could generate similar patterns of calling that are related to individuals' foraging strategies over the course of the day. Previous work (e.g. [9]) suggested that wintering songbirds have different foraging strategies in the morning and afternoon, and also reported an increase in flock size over the course of the day. Using experimental playbacks of recruitment calls, our current study demonstrates that tits will readily use vocal information to improve food detection at all times of the day: playing calls from blue tits, great tits, and marsh tits near bird feeders significantly reduced the latency to feeder discovery, facilitated recruitment of flock members, and increased the total number of birds attracted to food patches irrespective of the time of the day. The positive effect of broadcasting vocalizations on the recruitment of flock members was evident on the significantly higher number of individuals discovering the food source in the first 2 min after the original discovery, suggesting that it operates as an effective mechanism for increasing group size. By contrast, our results do not support the hypothesis that vocalizations are ignored in the afternoon owing to a switch in foraging strategies. The species-level analyses revealed almost identical results to the pooled analyses; all species recruited significantly faster under playback treatments in both the morning and in the afternoon (electronic supplementary material, figure S6). Thus, our experimental data support the framework we laid out in which individuals should make economic decisions (table 1).

Under the economic hypothesis, one reason for not making recruitment calls in the afternoon is that there are fewer potential flock members available to recruit in the local environment. While we found similar effects of the playback on the three response variables in the afternoon as in the morning, the latencies to feeder discovery were generally longer in afternoon trials, in both control and playback trials. This is likely to be because of individuals accumulating at alternative food sources over the course of the day (*sensu* [44]), resulting in a general decrease in movement between food patches later in the day (a pattern observed by [9]). However, as we have no indication that tits ignore vocalizations in the afternoon (as would be expected under the foraging strategies hypothesis), our findings suggest that recruitment calling could be less efficient at eliciting a response in the afternoon owing to a reduced pool of potential recruits. Thus, irrespective of the current group size, the benefits of recruiting, i.e. attracting group members, will be reduced relative to the risk of attracting a predator.

Many animals make signals in a social context, and recruitment calling is particularly interesting as it could be an important mechanism in underpinning fission–fusion dynamics, since individuals' decisions about when to recruit others has implications for group formation. However, it is not always clear what mechanisms underlie the decision to make a signal or not. We laid out a simple framework (table 1) that uses the concepts of optimality to make predictions about when recruitment calls should be made. This framework considers group size, and the impact of group size on competition and predation. Such an approach is powerful because the predictions can be tested relatively easily, and it provides a useful baseline on which future theoretical models can be built.

## Supplementary Material

Supplementary Information

## Data accessibility

Data and code used for analyses are available from the Dryad Digital Repository: https://doi.org/10.5061/dryad.21b52d0 [59].

## References

[1] Pravosudov VV, Grubb TC 1997 Energy management in passerine birds during the nonbreeding season. In Current ornithology (eds Nolan V, Ketterson ED, Thompson CF), vol. 14, pp. 189–234. Boston, MA: Springer.

[2] Reebs SG 2002 Plasticity of diel and circadian activity rhythms in fishes. Rev. Fish Biol. Fish. 12, 349–371. (10.1023/A:1025371804611)

[3] Lang SDJ, Mann RP, Farine DR In press Temporal activity patterns of predators and prey across broad geographic scales. Behav. Ecol. (10.1093/beheco/ary133)

[4] Gentle LK, Gosler AG 2001 Fat reserves and perceived predation risk in the great tit, *Parus major*. Proc. R. Soc. Lond. B 268, 487–491. (10.1098/rspb.2000.1405) PMC1088631 11296860

[5] Gregorini P 2012 Diurnal grazing pattern: its physiological basis and strategic management. Anim. Prod. Sci. 52, 416–430. (10.1071/AN11250)

[6] Owen DF 1954 The winter weights of titmice. Ibis 96, 299–309. (10.1111/j.1474-919X.1954.tb04130.x)

[7] Haftorn S 1992 The diurnal body weight cycle in titmice *Parus* spp. Ornis Scand. 23, 435–443. (10.2307/3676674)

[8] Gosler AG, Greenwood JJD, Perrins C 1995 Predation risk and the cost of being fat. Nature 377, 621–623. (10.1038/377621a0)

[9] Farine DR, Lang SDJ 2013 The early bird gets the worm: foraging strategies of wild songbirds lead to the early discovery of food sources. Biol. Lett. 9, 20130578 (10.1098/rsbl.2013.0578) 24108676 PMC3871345

[10] Danchin E, Giraldeau L-A, Valone TJ, Wagner R.H 2004 Public information: from nosy neighbors to cultural evolution. Science 305, 487–491. (10.1126/science.1098254) 15273386

[11] Dall SRX, Giraldeau L-A, Olsson O, McNamara JM, Stephens DW 2005 Information and its use by animals in evolutionary ecology. Trends Ecol. Evol. 20, 187–193. (10.1016/j.tree.2005.01.010) 16701367

[12] Giraldeau LA, Valone TJ, Templeton JJ 2002 Potential disadvantages of using socially acquired information. Phil. Trans. R. Soc. Lond. B 357, 1559–1566. (10.1098/rstb.2002.1065) 12495513 PMC1693065

[13] Galef BG 2009 Strategies for social learning: testing predictions from formal theory. Adv. Study Behav. 39, 117–151. (10.1016/S0065-3454(09)39004-X)

[14] Baciadonna L, McElligott AG, Briefer EF 2013 Goats favour personal over social information in an experimental foraging task. PeerJ 1, e172 (10.7717/peerj.172) 24109556 PMC3792185

[15] Pruitt JN, Wright CM, Keiser CN, DeMarco AE, Grobis MM, Pinter-Wollman N 2016 The Achilles’ heel hypothesis: misinformed keystone individuals impair collective learning and reduce group success. Proc. R. Soc. B 283, 20152888 (10.1098/rspb.2015.2888) PMC4795039 26817771

[16] Laland KN 2004 Social learning strategies. Learn. Behav. 32, 4–14. (10.3758/BF03196002) 15161136

[17] Kendal RL, Coolen I, Laland KN 2004 The role of conformity in foraging when personal and social information conflict. Behav. Ecol. 15, 269–277. (10.1093/beheco/arh008)

[18] van de Waal E, Borgeaud C, Whiten A 2013 Potent social learning and conformity shape a wild primate's foraging decisions. Science 340, 483–485. (10.1126/science.1232769) 23620053

[19] Aplin LM, Farine DR, Morand-Ferron J, Cockburn A, Thornton A, Sheldon BC 2015 Experimentally induced innovations lead to persistent culture via conformity in wild birds. Nature 518, 538–541. (10.1038/nature13998) 25470065 PMC4344839

[20] Clay Z, Smith CL, Blumstein DT 2012 Food-associated vocalizations in mammals and birds: what do these calls really mean? Anim. Behav. 83, 323–330. (10.1016/j.anbehav.2011.12.008)

[21] Lima SL 1995 Back to the basics of anti-predatory vigilance: the group-size effect. Anim. Behav. 491, 11–20. (10.1016/0003-3472(95)80149-9)

[22] Goodale E, Kotagama SW 2008 Response to conspecific and heterospecific alarm calls in mixed-species bird flocks of a Sri Lankan rainforest. Behav. Ecol. 19, 887–894. (10.1093/beheco/arn045)

[23] Hauser MD, Marler P 1993 Food-associated calls in rhesus macaques (*Macaca mulatta*): I. Socioecological factors. Behav. Ecol. 4, 194–205. (10.1093/beheco/4.3.194)

[24] Judd TM, Sherman PW 1996 Naked mole-rats recruit colony mates to food sources. Anim. Behav. 52, 957–969. (10.1006/anbe.1996.0244)

[25] Evans CS, Marler P 1994 Food calling and audience effects in male chickens, *Gallus gallus*: their relationships to food availability, courtship and social facilitation. Anim. Behav. 47, 1159–1170. (10.1006/anbe.1994.1154)

[26] Van Krunkelsven E, Dupain J, Van Elsacker L, Verheyen RF 1996 Food calling in captive bonobos (*Pan paniscus*): an experiment. Int. J. Primatol. 17, 207–217. (10.1007/BF02735448)

[27] Heinrich B 1988 Winter foraging at carcasses by three sympatric corvids, with emphasis on recruitment by the raven, *Corvus corax*. Behav. Ecol. Sociobiol. 23, 141–156. (10.1007/BF00300349)

[28] Wilkinson GS, Boughman JW 1998 Social calls coordinate foraging in greater spear-nosed bats. Anim. Behav. 55, 337–350. (10.1006/anbe.1997.0557) 9480702

[29] Elgar MA 1986 House sparrows establish foraging flocks by giving chirrup calls if the resources are divisible. Anim. Behav. 34, 169–174. (10.1016/0003-3472(86)90020-5)

[30] Shalter MD 1978 Localization of passerine seeet and mobbing calls by goshawks and pygmy owls. Z. Tierpsychol. 59, 338–350.

[31] Leech SM, Leonard ML 1997 Begging and the risk of predation in nestling birds. Behav. Ecol. 8, 644–646. (10.1093/beheco/8.6.644)

[32] Krams I 2001 Communication in crested tits and the risk of predation. Anim. Behav. 61, 1065–1068. (10.1006/anbe.2001.1702)

[33] Hamilton WD 1971 Geometry for the selfish herd. J. Theor. Biol. 31, 295–311. (10.1016/0022-5193(71)90189-5) 5104951

[34] Schneider KJ 1984 Dominance, predation, and optimal foraging in white-throated sparrow flocks. Ecology 65, 1820–1827. (10.2307/1937778)

[35] Cresswell W, Quinn JL 2011 Predicting the optimal prey group size from predator hunting behaviour. J. Anim. Ecol. 80, 310–319. (10.1111/j.1365-2656.2010.01775.x) 21244418

[36] Dostie MJ, Lusseau D, Bonnell T, Clarke PM, Chaplin G, Kienzle S, Barrett L, Henzi SP 2016 Proof of principle: the adaptive geometry of social foragers. Anim. Behav. 119, 173–178. (10.1016/j.anbehav.2016.07.011)

[37] Milligan ND, Radersma R, Cole EF, Sheldon BC 2017 To graze or gorge: consistency and flexibility of individual foraging tactics in tits. J. Anim. Ecol. 86, 826–836. (10.1111/1365-2656.12651) 28191628

[38] Farine DR, Garroway CJ, Sheldon BC 2012 Social network analysis of mixed-species flocks: exploring the structure and evolution of interspecific social behaviour. Anim. Behav. 84, 1271–1277. (10.1016/j.anbehav.2012.08.008)

[39] Farine DR, Aplin LM, Sheldon BC, Hoppitt W 2015 Interspecific social networks promote information transmission in wild songbirds. Proc. R. Soc. B 282, 20142804 (10.1098/rspb.2014.2804) PMC4345451 25673683

[40] Farine DR et al. 2015 The role of social and ecological processes in structuring animal populations: a case study from automated tracking of wild birds. R. Soc. open sci. 2, 150057 (10.1098/rsos.150057) 26064644 PMC4448873

[41] Firth JA, Sheldon BC, Farine DR 2016 Pathways of information transmission among wild songbirds follow experimentally imposed changes in social foraging structure. Biol. Lett. 12, 20160144 (10.1098/rsbl.2016.0144) 27247439 PMC4938043

[42] Suzuki TN, Kutsukake N 2017 Foraging intention affects whether willow tits call to attract members of mixed-species flocks. R. Soc. open sci. 4, 170222 (10.1098/rsos.170222) 28680675 PMC5493917

[43] Aplin LM, Farine DR, Morand-Ferron J, Sheldon BC 2012 Social networks predict patch discovery in a wild population of songbirds. Proc. R. Soc. B 279, 4199–4205. (10.1098/rspb.2012.1591) PMC3441092 22915668

[44] Farine DR, Aplin LM, Garroway CJ, Mann RP, Sheldon BC 2014 Collective decision making and social interaction rules in mixed-species flocks of songbirds. Anim. Behav. 95, 173–182. (10.1016/j.anbehav.2014.07.008) 25214653 PMC4157325

[45] Carlson NV, Healy SD, Templeton CN 2017 A comparative study of how British tits encode predator threat in their mobbing calls. Anim. Behav. 125, 77–92. (10.1016/j.anbehav.2017.01.011)

[46] Ficken MS 1981 Food finding in black-capped chickadees: altruistic communication? Wilson Bull. 93, 393–394.

[47] Mahurin EJ, Freeberg TM 2009 Chick-a-dee call variation in Carolina chickadees and recruiting flockmates to food. Behav. Ecol. Sociobiol. 20, 111–116. (10.1093/beheco/arn121)

[48] Suzuki TN 2012 Long-distance calling by the willow tit, *Poecile montanus*, facilitates formation of mixed-species foraging flocks. Ethology 11, 10–16. (10.1111/j.1439-0310.2011.01982.x)

[49] Hinde RA 1952 The behaviour of the great tit (*Parus major*) and some other related species. Behaviour 2, 1–201.

[50] Audacity Team 1999 Audacity: free audio editor and recorder, version 2.1.0., accessed 5 March 2015. See www.audacityteam.org/.

[51] Boersma P, Weenick D 2016 Praat: doing phonetics by computer, version 6.0.17., accessed 21 April 2016. See www.praat.org/.

[52] Farine DR 2017 A guide to null models for animal social network analysis. Methods Ecol. Evol. 8, 1309–1320. (10.1111/2041-210X.12772) 29104749 PMC5656331

[53] Lo S, Andrews S 2015 To transform or not to transform: using generalized linear mixed models to analyse reaction time data. Front. Psychol. 6, 1171. 26300841 10.3389/fpsyg.2015.01171 PMC4528092

[54] Engqvist L 2005 The mistreatment of covariate interaction terms in linear model analyses of behavioural and evolutionary ecology studies. Anim. Behav. 70, 967–971. (10.1016/j.anbehav.2005.01.016)

[55] R Core Team. 2014 R: a language and environment for statistical computing. Vienna, Austria: R Foundation for Statistical Computing See http://www.R-project.org/.

[56] Bates D, Mächler M, Bolker B, Walker S 2015 Fitting linear mixed-effects models using lme4. J. Stat. Softw. 67, 1–48. (10.18637/jss.v067.i01)

[57] Chapman CA, Lefebvre L 1990 Manipulating foraging group size: spider monkey food calls at fruiting trees. Anim. Behav. 39, 891–896. (10.1016/S0003-3472(05)80953-4)

[58] Association for the Study of Animal Behaviour (ASAB). 2012 Guidelines for the treatment of animals in behavioural research and teaching. Anim. Behav. 83, 301–309. (10.1016/j.anbehav.2011.10.031)

[59] Hillemann F, Cole EF, Keen SC, Sheldon BC, Farine DR 2019 Data from: Diurnal variation in the production of vocal information about food supports a model of social adjustment in wild songbirds Dryad Digital Repository. (10.5061/dryad.21b52d0). PMC6408885 30963842

