# Supplementary material for: Diurnal variation in the production of vocal information about food supports a model of social adjustment in wild songbirds
Source: Proc Biol Sci. 2019 Feb 27;286(1897):20182740. doi: 10.1098/rspb.2018.2740 (PMC6408885; doi:10.1098/rspb.2018.2740)
Supplement: Supplementary Information [file rspb20182740supp1.pdf]

Supplementary Information

Diurnal variation in the production of vocal information about food supports a model of social adjustment in wild songbirds

F. Hillemann, E. F. Cole, S. C. Keen, B. C. Sheldon, D. R. Farine

Proceedings of the Royal Society B: Biological Sciences
DOI: 10.1098/rspb.2018.2740

Composition of playback stimuli

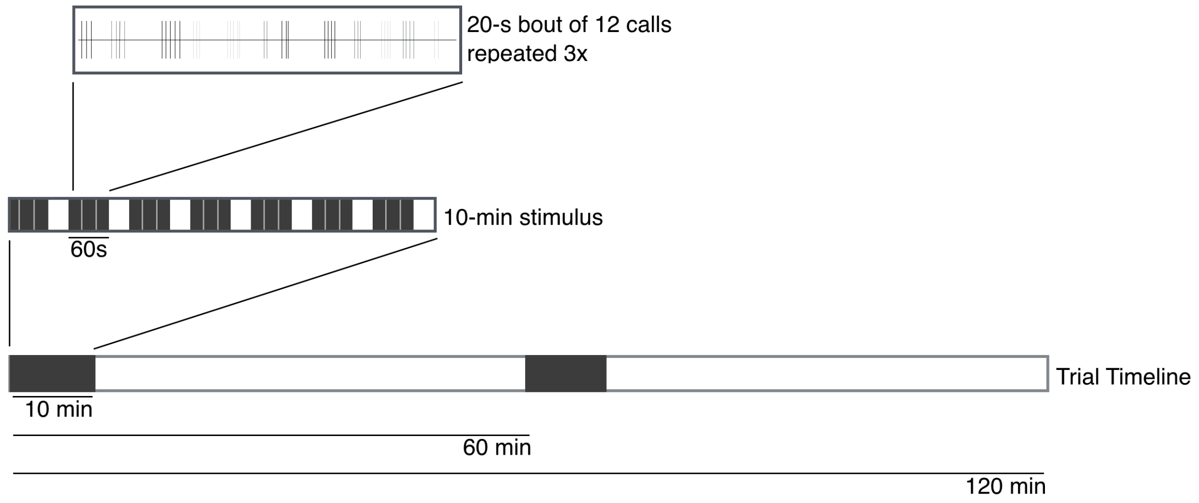

Figure S1. Schematic timeline of playback trials. For each stimulus, we combined four calls from each of the three species to a 20-s calling sequence with a random succession of calls. This 20-s sequences was repeated three times, creating a one-minute bout of calls. Separated by 30-s periods of white noise, the one-minute bout was repeated seven times to create a 10-min stimulus. White areas represent silent periods.

Vocal behaviour at feeders: call rate

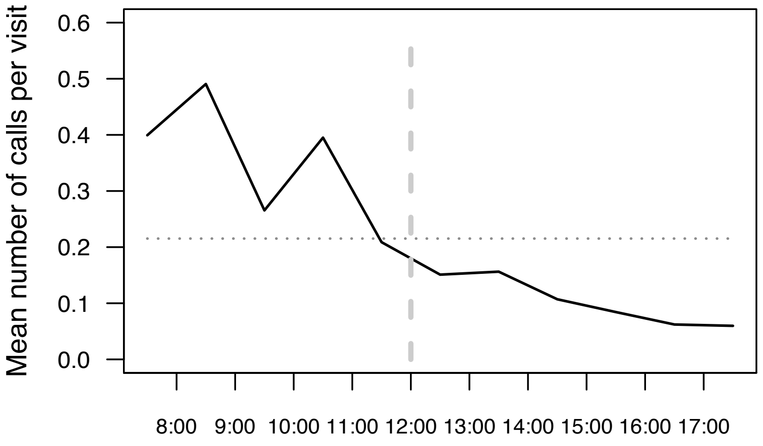

Figure S2. Hourly call rates measured as number of calls per feeder visit. The mean number of calls per visit declined throughout the day. The black line is the observed calling rate (n=18 trials), the dotted horizontal line is the expected calling rate. The calling rate before 11:00 h is always higher than the random rate and the mean value in all afternoon hours.

Vocal behaviour at feeders: number of notes and number of feeder visits

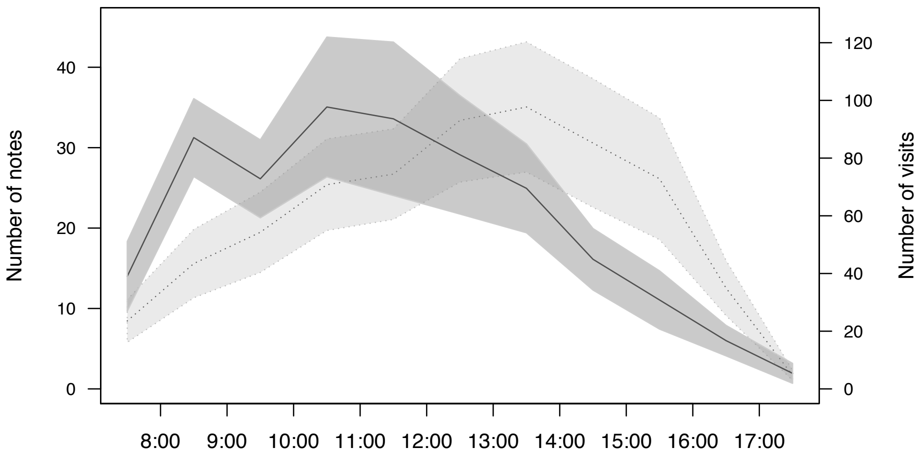

Figure S3. Diurnal patterns at feeders. Vocal activity at the feeder measured as sum of notes of all calls (solid line), and foraging activity measured as hourly mean number of feeder visits (dotted line); shaded areas are standard errors of respective means, n=18 trials. Production of acoustic information decreases into the afternoon, both when measured as number of calls (Figure 2 in the main text) and number of notes per hour.

Vocal behaviour at feeders: call type pattern

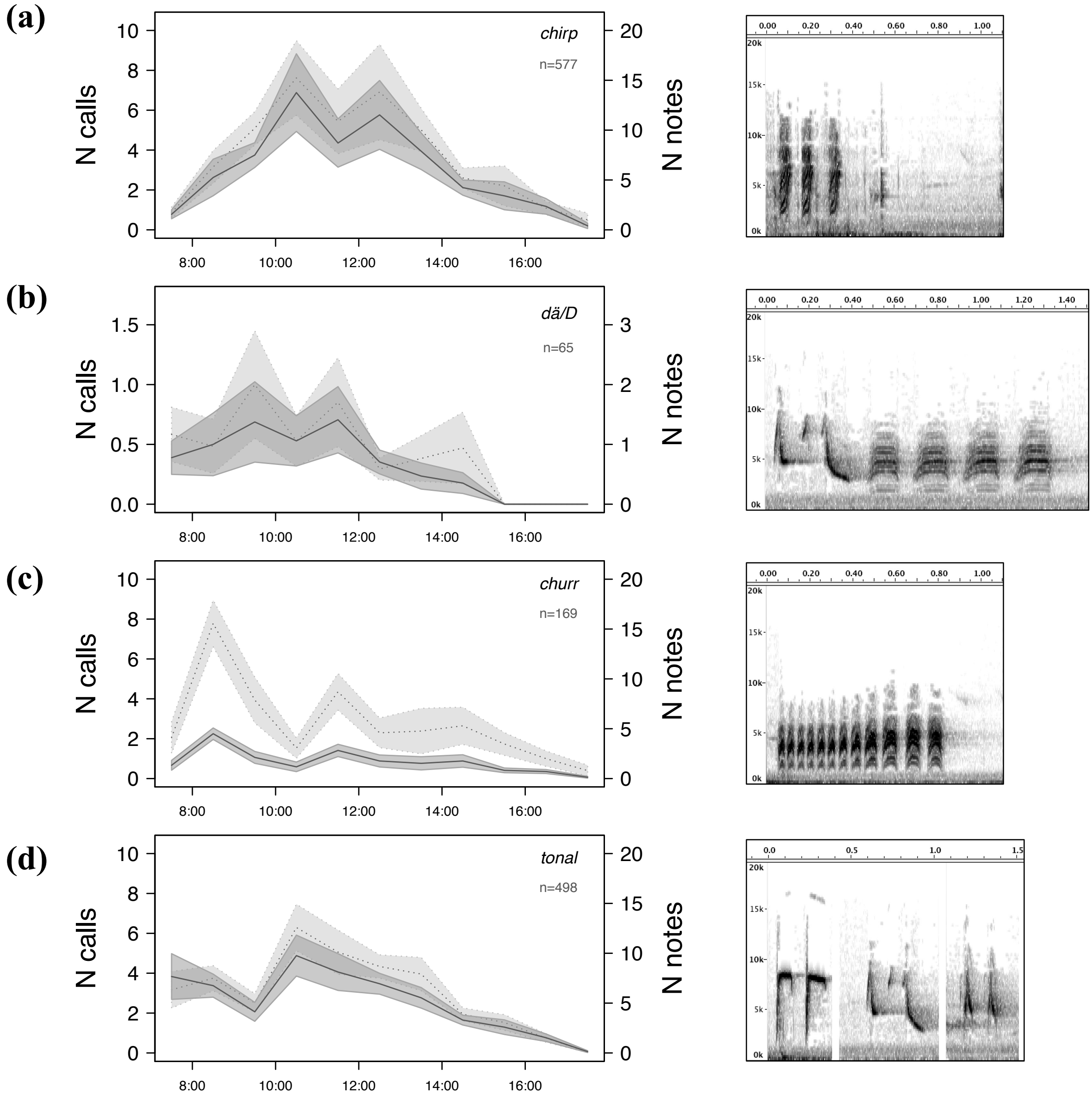

Figure S4. Main call types recorded at feeding stations: temporal pattern and spectrograms. Mean number of calls (solid line) and notes (dotted line) recorded per hour and standard errors (shaded area) across all 18 trials for (a) *chirp* calls, (b) *dä/D*-calls, (c) *churr* calls, and (d) calls only consisting of tonal elements. Total number of calls recorded: 577 *chirp* calls, 65 *dä/D* calls, 169 *churr* calls, 498 *tonal* calls. For classification and naming of call types, we followed a recent overview of call types in British tits (Carlson et al. 2017). An audio file with example calls of each call type is available from the Dryad Digital Repository: http://dx.doi.org/10.5061/dryad.21b52d0.

For each plot, the y-axes are in a ratio of 1:2 (number of calls: number of notes). Only the *churr* call, which was not used in the playback, shows a marked variation in number of notes per call throughout the day: *churr* calls given in the early morning hours had more notes than *churr* calls given in the afternoon.

Number of notes in chirp and dä/D calls (data from Carlson et al. 2017)

Table S1: Number of notes per *chirp* and *dä/D* call in different contexts. Mean number of *chirp* notes and mean number of *dä/D* notes in blue tit, great tit, and marsh tit calls, given with standard errors and sample sizes in brackets, recorded during presentation of robotic stuffed taxidermy mounts. Data underlying Carlson, N. V., Healy, S. D., & Templeton, C. N. 2017. A comparative study of how British tits encode predator threat in their mobbing calls. Animal Behaviour, 125, 77-92, DOI: 10.1016/j.anbehav.2017.01.011, and was kindly provided by Nora Carlson.

	**Blue tit** ***chirp* calls**	**Great tit** ***chirp* calls**	**Marsh tit** ***dä/D* calls**
Non-threat control (Grey partridge, *Perdix perdix*)	1.72 ± 0.15 (124 calls, 41 trials)	0.59 ± 0.10 (36 calls, 35 trials)	0.85 ± 0.30 (12 calls, 7 trials)
High-threat predator (Sparrowhawk, *Accipiter nisus*)	0.50 ± 0.04 (191 calls, 43 trials)	0.04 ± 0.01 (24 calls, 42 trials)	5.22 ± 0.33 (158 calls, 9 trials)

Independence of trials

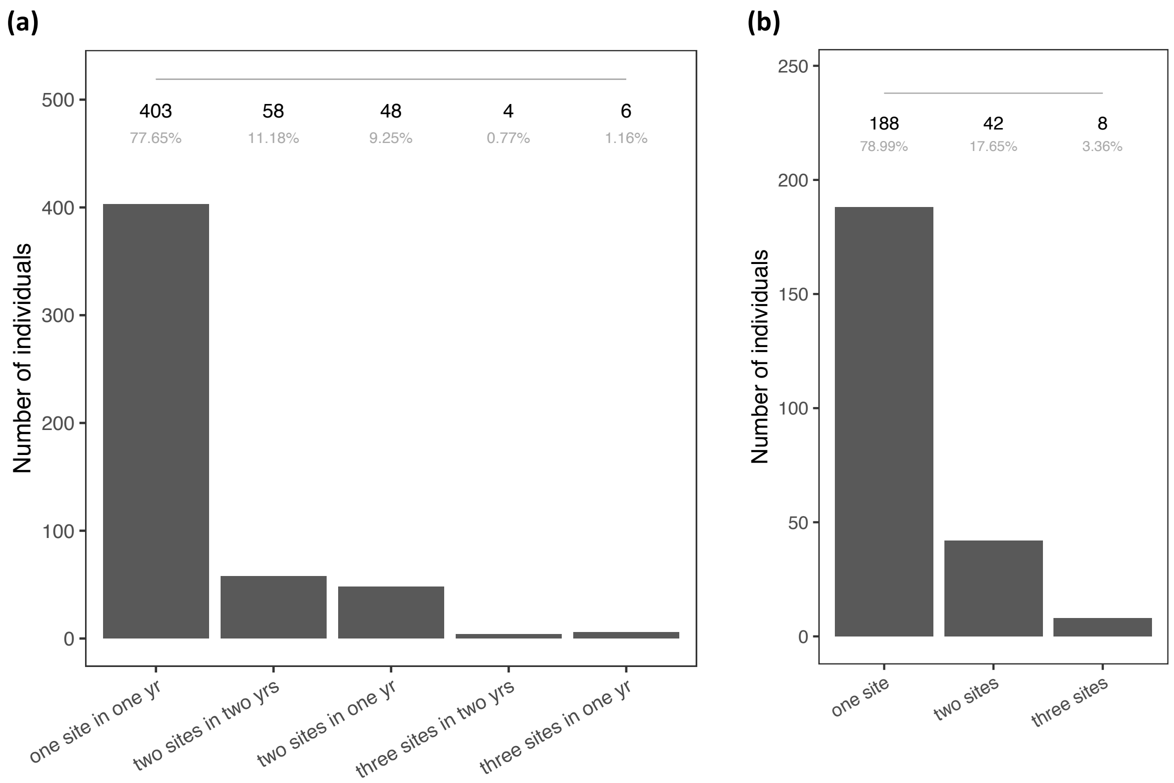

Figure S5. Number of individuals that were recorded at one or multiple sites in (a) feeder discovery trials and (b) playback experiments. Feeder discovery trials were conducted at 18 unique sites, in two consecutive winters (10 sites in early 2016 and 8 sites in early 2017). In (a), bars represent the number of individuals that were recorded at only one site, at two sites but once in 2016 and once 2017, at two sites within the same year, or three times in total. The playback experiment was conducted at twelve sites across the woods. In (b), bars represent the number of individuals that were recorded at either one, two, or three different sites. The total number of individuals recorded in the experiment is indicated by the grey line above the bars, black numbers give the total number of individuals recorded per category, percentages are given in grey.

The majority of individuals were recorded at only one site for both feeder discovery trials and playback experiments. Across the 18 feeder discovery trials, most individuals were recorded either at only one site throughout the study, or at two sites in total but only one in 2016 and 2017 each (89%, or 461 of 519 unique individuals recorded in total fall into these two categories). Across the 12 sites used for the playback experiment, most individuals were recorded at only one site (79%, or 188 of 238 individuals recorded in total). From those individuals that visited more than one playback site, only about half of the birds experienced the same condition twice (24 of the 50 birds that were recorded at more than one site; number of), and no individual experienced the same experimental condition more than twice.

Response to playbacks: species-level pattern

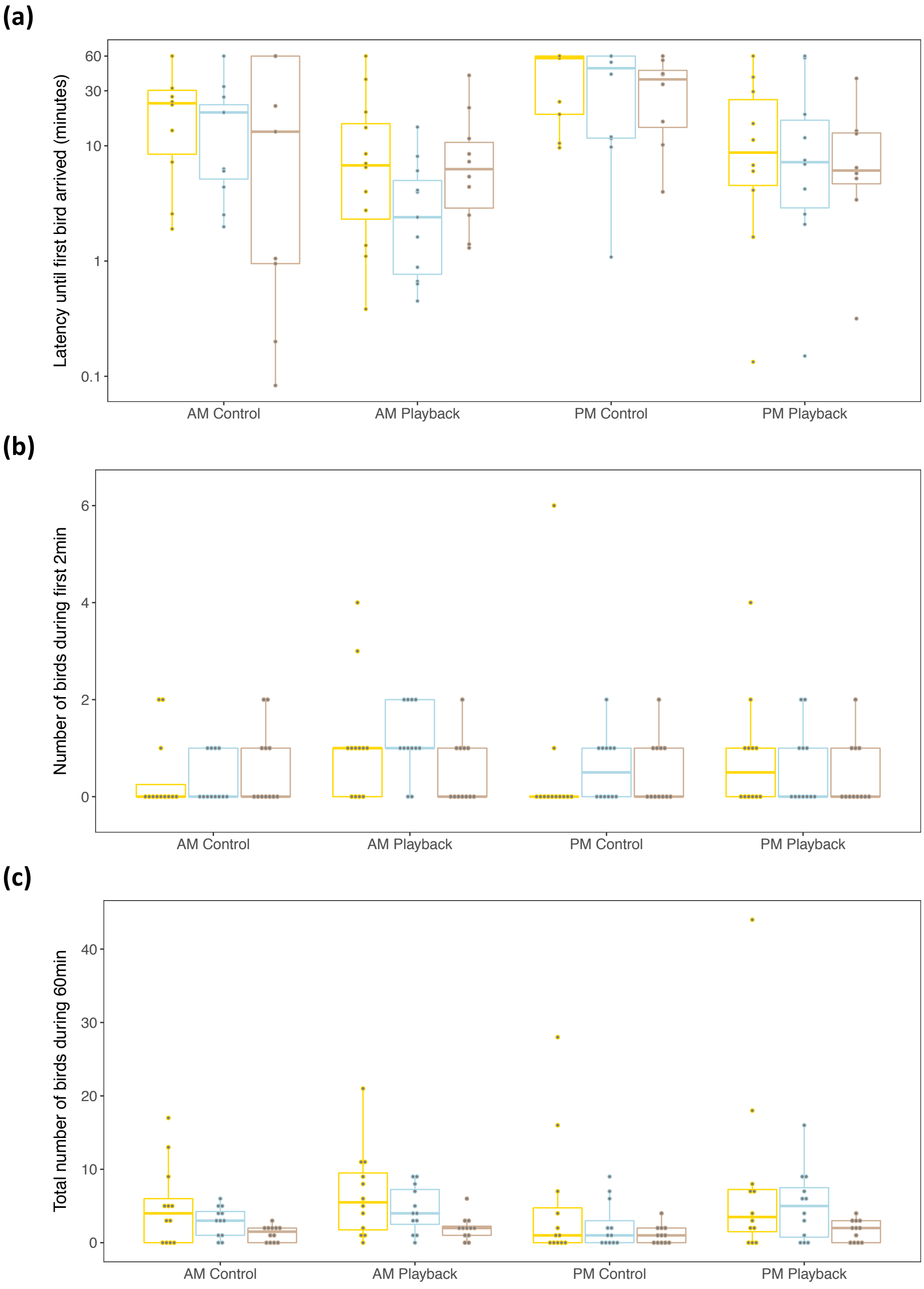

Figure S6. Response to the four experimental treatments, measured as (a) latency to feeder discovery (note log scale), (b) recruitment to feeder, and (c) number of birds visiting during the one-hour long trial. Data points from individual trials, with boxplots showing median and interquartile range (IQR), and whiskers represent 1.5 x IQR. Results are presented for the four experimental treatment conditions, and separately for each species, as indicated by colours (yellow: great tit, blue: blue tit, brown: marsh tit).

Table S2. Results of GLMMs analysing the response to playback trials separately for each species. Estimated effects of fixed factors (time of day: PM relative to AM, treatment: playback relative to silent control, and order of trial at a given site: 1st, 2nd, 3rd, or 4th) on the three measures describing the response to playback trials. Experimental site was included as a random term in all models. Model outputs provided are: degrees of freedom (df), coefficient and standard error (SE), z-statistic, and standard P-value. Significant terms (P-value < 0.05) are indicated in bold.

**Species**	**Response Variable ~ Fixed Factors**	**df**	**Coefficient ± SE**	***z***	***P***
Great Tit	Latency First Bird				
	Time of Day	1	0.42 ± 0.31	1.36	0.172
	**Treatment**	**1**	**-0.92 ± 0.32**	**-2.89**	**0.004**
	**Order of Trial**	**1**	**-0.32 ± 0.14**	**-2.22**	**0.026**
	Recruitment				
	Time of Day	1	-0.20 ± 0.36	-0.56	0.572
	**Treatment**	**1**	**0.76 ± 0.37**	**2.06**	**0.039**
	**Order of Trial**	**1**	**0.38 ± 0.17**	**2.26**	**0.024**
	Number of Birds				
	Time of Day	1	0.03 ± 0.12	0.23	0.815
	**Treatment**	**1**	**0.52 ± 0.12**	**4.22**	**< 0.001**
	**Order of Trial**	**1**	**0.35 ± 0.06**	**6.22**	**< 0.001**
Blue Tit	Latency First Bird				
	**Time of Day**	**1**	**1.02 ± 0.28**	**3.59**	**< 0.001**
	**Treatment**	**1**	**-1.39 ± 0.27**	**-5.13**	**< 0.001**
	**Order of Trial**	**1**	**-0.33 ± 0.13**	**-2.52**	**0.011**
	Recruitment				
	Time of Day	1	-0.25 ± 0.36	-0.70	0.481
	Treatment	1	0.65 ± 0.37	1.74	0.082
	Order of Trial	1	0.18 ± 0.16	1.10	0.270
	Number of Birds				
	Time of Day	1	0.00 ± 0.15	0.20	0.984
	**Treatment**	**1**	**0.60 ± 0.15**	**3.85**	**< 0.001**
	**Order of Trial**	**1**	**0.21 ± 0.07**	**3.12**	**0.002**
Marsh Tit	Latency First Bird				
	Time of Day	1	0.22 ± 0.39	0.56	0.576
	**Treatment**	**1**	**-1.00 ± 0.39**	**-2.60**	**0.009**
	Order of Trial	1	-0.18 ± 0.19	-0.96	0.338
	Recruitment				
	Time of Day	1	-0.16 ± 0.41	-0.39	0.696
	Treatment	1	-0.20 ± 0.42	-0.49	0.623
	**Order of Trial**	**1**	**0.38 ± 0.19**	**1.98**	**0.047**
	Number of Birds				
	Time of Day	1	-0.16 ± 0.24	-0.70	0.486
	Treatment	1	0.44 ± 0.24	1.82	0.069
	**Order of Trial**	**1**	**0.22 ± 0.11**	**2.04**	**0.041**

The three species show similar behavioural responses to the four experimental treatments. Compared to silent control trials, feeders were discovered earlier and by more individuals during playback trials, and the effect of playback was independent of time of the day (AM or PM). Note that the three species differ in their relative abundance across the population, and different numbers of individuals per species were recorded at experimental sites: mean number of great tits: 24.4 ± 7.4 SE individuals, blue tits: 14.8 ± 2.9 SE individuals, marsh tits: 6 ± 0.9 SE individuals. Too few observations of the less abundant species might not allow to properly investigate effects on number of individuals.
